# Mediators of Interorgan Crosstalk in Metabolic Inflammation

**DOI:** 10.1155/2013/609096

**Published:** 2013-09-04

**Authors:** Massimo Collino, Assaf Rudich, Daniel Konrad

**Affiliations:** ^1^Department of Drug Science and Technology, University of Turin, via Pietro Giuria 9, 10125 Torino, Italy; ^2^Department of Clinical Biochemistry and Pharmacology, Faculty of Health Sciences and the National Institute of Biotechnology in the Negev, Ben-Gurion University of the Negev, 84103 Beer-Sheva, Israel; ^3^Division of Paediatric Endocrinology and Diabetology, University Children's Hospital, 8032 Zurich, Switzerland

The concept proposing a major role for interorgan crosstalk as a link between dysmetabolism and inflammation largely builds on two major conceptual advances that developed mainly in the last two decades. The first one is the recognition that endocrine-type crosstalk exists between virtually all organs and tissues, extending way beyond very specific, “classical” endocrine glands. This has relied on various lines of experimental approaches, including “omics” types of data, revealing that a large fraction of the expressed genome of many tissues encodes known and predicted secreted proteins. Complementarily, tissue-specific genetic manipulations provided *in vivo* evidence for the ability to alter the function of a tissue distant from the site directly affected by the genetic intervention. The second conceptual advance is the huge expansion in our understanding of the homeostatic-physiological roles, as well as the pathophysiological involvement, of the immune system in diverse types of diseases. In this context, two decades ago, the finding that tumour necrosis factor-*α* (TNF-*α*) is overexpressed in adipose tissue of obese mice provided the first clear link between obesity and chronic inflammation [1]. Almost at the same time, the discovery of leptin, the first adipokine that signals through a cytokine receptor family member, sparked the understanding that inflammatory changes within adipose tissue may alter the way this tissue communicates with various organs, even the brain [2]. More recently, a wealth of data established the concept that obesity is associated with systemic and tissue-level low-grade chronic inflammation, initially recognized by adipose tissue infiltration with inflammatory cells in response to an excessive energetic nutrient load [3, 4]. The concept of “metabolic inflammation” or “metaflammation” was eventually formulated and fueled by examples of specific mechanisms tying metabolic mediators to inflammatory cascades and inflammatory mediators to alterations in metabolic regulation [5]. The metabolic inflammation, possibly initiated in response to excess calories/high fat feeding in the adipose tissue, may subsequently involve other metabolic organs such as the liver, skeletal muscle, and pancreas, contributing to metabolic dysfunction and insulin resistance. This multiorgan involvement of obesity-associated inflammation presents a challenge to researchers attempting to clarify the complex signaling mechanisms of the interorgan crosstalk leading to metabolic dysfunction. Alongside inflammation pertaining to “metabolic” organs, studies published during the past decade have convincingly demonstrated a pathophysiological role for the inflammatory response in the development of cardiovascular disease (CVD), the most important complication of the metabolic syndrome and diabetes [6]. In addition, a multitude of mediators of metabolic inflammation have been found to modulate vascular homeostasis, suggesting further mechanisms of interorgan crosstalk between metabolic tissues and the vasculature. Therefore, the low chronic inflammatory state may be responsible for both insulin resistance and endothelial dysfunction, providing deleterious connections between inflammation and metabolic/CVD processes.

This special issue aims to highlight selected topics projecting on advances in our current understanding of the players involved in interorgan communication in obesity-associated metabolic diseases. 

In their review, S. Raschke and J. Eckel present an update on bioactive proteins secreted by adipose tissue and skeletal muscles (termed “adipokines” and “myokines”, resp.), revealing their peculiar interplay, which represents a yin/yang-type balance: adipokines, upregulated in the obese state, tend to contribute to establish a chronic inflammatory environment that promotes pathological processes such as atherosclerosis and insulin resistance, whereas myokines seem to largely counteract these effects of adipokines, likely reflecting their essential regulatory role in muscle metabolism during contraction. Yet, as more than half of the described myokines are also secreted by adipocytes, the authors suggest to term these cytokines “adipo-myokines,” whose beneficial or adverse effects are strictly dependent on their local concentration and kinetics. 

A confirmation of this general principle can be found in the review by T. Romacho and colleagues describing the cellular effects of the recently identified adipokine Visfatin/Nampt. Within the adipose tissue, Visfatin/Nampt is not only synthesized and released by adipocytes but also by inflammatory cells, like activated macrophages. Very recently, Visfatin/Nampt expression has been reported in cardiomyocytes, hepatocytes, and myoblasts, thus suggesting its potential role in an even more intricate interorgan metabolic crosstalk. The authors summarize convincing evidence for its deleterious effects on the cardiovascular system, including monocyte/macrophage activation, vascular inflammation, and remodeling. At the same time, the authors quote several papers on beneficial actions of Visfatin/Nampt in ischemia-related clinical conditions.

In their research article, R. Schlich and colleagues investigate the role of vascular endothelial growth factor (VEGF). This well-known growth factor is also released from visceral adipose tissue in obesity and from perivascular adipose tissue of patients with type 2 diabetes and may constitute yet another interorgan mediator, in this instance, linking adipose tissue inflammation to abnormal vascular smooth muscle cells (VSMC) proliferation. Using a model of VSMC proliferation induced by adipocyte-conditioned medium (CM), the authors clearly show that this effect was mainly mediated by VEGF. Moreover, they demonstrate that CM also induced the release of VEGF by VSMC, which in turn could stimulate proliferation via “second-level” autocrine/paracrine communication.

Another immune system (complement-related) mediator, adipose-derived factor with potential autocrine/paracrine and endocrine functions is the acylation stimulating protein (ASP). Using an *in vivo* model of diet-induced obesity, which mimics a typical unhealthy western diet with both high fat and high sucrose intakes, A. Fisette and colleagues demonstrate the paradoxical effects of acute administration of ASP. Particularly, they show that ASP supplementation improved glucose clearance through a short-term insulin sensitizing process in spite of its proinflammatory properties, as demonstrated by increased serum and tissue levels of IL-6 and TNF-*α*. This may represent a growing current notion expanding our view of the links between inflammation and metabolic regulation [7].

E. Benetti and colleagues focus their attention on the deleterious effects of high sugar intake. They demonstrated that mice chronically fed a sugar-enriched diet develop dyslipidemia and hyperinsulinemia. These metabolic effects were associated with increased infiltration of inflammatory cells into skeletal muscle. Most notably, dyslipidemia and hyperinsulinemia could be prevented by oral administration of a selective agonist of the nuclear receptor peroxisome proliferator activated receptor-*δ*, which positively correlated with increased muscular levels of the myokine FGF-21, a mediator with expanding roles in metabolic and energy regulation [8]. 

The role of diet components/additives in affecting subclinical inflammation and insulin resistance is further investigated by S. Bhattacharyya and colleagues. They provide novel insights into the mechanisms of carrageenan-induced inflammation. Interestingly, mice lacking Bcl10, a key molecule in the activation of the inflammatory cascade in epithelial and immune cells, were partly protected from carrageenan-induced inflammation, and such effect was related to an inhibition of both canonical and noncanonical pathways of NF-*κ*B activation. 

A potential intestinal participation in the pathogenesis of insulin resistance is more widely addressed in the review by B. M. Carvalho and colleagues, showing how gut microbiota modulation promoted by a bacterial diversity shift mediated by overnutrition increases intestinal permeability, culminating in inflammation and insulin resistance. Herein, activation of inflammatory pathways by gut-derived bacteria products and metabolites is discussed, with particular reference to the key role of the inflammasome multiprotein complex. This recently identified novel player of the intercellular crosstalk linking chronic inflammation to metabolic disease pathogenesis is also the main topic of the review article by B. Benetti et al.

The role of selective inflammatory mediators in pancreatic *β*-cell dysfunction has been investigated in two different research articles. L. Quintana-Lopez and colleagues demonstrate in cultured islet *β*-cells that nitric oxide (NO) is an important mediator of the antiproliferative effect induced by proinflammatory cytokines, such as IL-1*β*, IFN-*γ*, and TNF-*α*. In keeping with these *in vitro* data, the same authors report that early treatment of biobred rats (inbred laboratory rats spontaneously developing autoimmune type 1 diabetes) with the NO synthase inhibitor L-NMMA improved *β*-cell proliferation compared to untreated animals. In addition, A. Puddu and colleagues demonstrate that the detrimental effects evoked by exposing the hamster pancreatic *β*-cell line HIT-T15 to a pool of advanced glycation end products are prevented by cell treatment with the incretin hormone glucagon-like peptide-1 (GLP-1), which was shown here to restore selective pathways regulating proinsulin production. 

Two additional articles discuss the molecular mechanisms underlying the positive association between obesity-associated insulin resistance and cardiac hypertrophy or destructive periodontal disease. Specifically, M. Asrih and colleagues report evidence on the dual role of the cytosolic signaling proteins mitogen-activated protein kinases in inducing synthesis of both specific mediators of cardiac hypertrophy (such as the gap junction protein connexin 43 and the cardiotrophin-1) and glucose transporters (by affecting the insulin signaling pathway) in cardiomyocytes, thus suggesting selective intracellular mechanisms of cardiac adverse remodeling associated with insulin resistance. R. Khosravi and colleagues offer in their overview potential explanations for the higher risk of destructive periodontal disease in obesity and insulin resistance; that is, they provide evidence for TNF-*α* and IL-6 as crucial inflammatory mediators connecting these different diseases.

Finally, R. Murphy and colleagues propose an intriguing approach to better elucidate the firm cause-effect relationships between obesity and insulin resistance development. In fact, although obesity is often reported to confer the highest risk for the development of metabolic diseases, the key events in this process are difficult to disentangle from compensatory effects and epiphenomena. The authors of this review support the suggestion that a deep investigation on key monogenic disorders may significantly contribute to unravel the pathogenic links between the defined molecular defect and the related exaggerated metabolic disorders, without the confounding effects of all the other components. For instance, they show how the key role of components of pancreatic *β*-cell function can be confirmed by evidence, demonstrating that common monogenic pancreatic *β*-cell defects lead to early onset of diabetes even in the absence of obesity-related insulin resistance.

Overall, we hope that readers will find the articles in this issue not only interesting but also instructive. They might even serve as a source of inspiration for future investigations aiming to further elucidate the intricate inflammatory mechanisms operational in the pathogenesis, and potentially also the resolution, of obesity-associated insulin resistance and type 2 diabetes. Such lines of research may raise hope for discovering new strategies to battle one of the key drivers of human morbidity in modern sedentary times.

*Massimo Collino* *Massimo Collino* *Assaf Rudich* *Assaf Rudich* *Daniel Konrad* *Daniel Konrad*
